# Extent of Endoscopic Sinus Surgery for Odontogenic Sinusitis of Endodontic Origin with Ethmoid and Frontal Sinus Involvement

**DOI:** 10.3390/jcm13206204

**Published:** 2024-10-18

**Authors:** Marta Aleksandra Kwiatkowska, Kornel Szczygielski, Dariusz Jurkiewicz, Piotr Rot

**Affiliations:** Department of Otolaryngology and Oncological Laryngology with Division of Cranio-Maxillo-Facial Surgery, Military Institute of Medicine—National Research Institute, 04-141 Warsaw, Poland

**Keywords:** odontogenic sinusitis, maxillary antrostomy, frontal surgery, endoscopic sinus surgery, periapical lesion

## Abstract

**Background/Objectives**: Odontogenic sinusitis (ODS) is the most common cause of unilateral maxillary sinus opacification. Initial treatment consists of intranasal steroids and antimicrobial therapy. In case of persistence of the disease, endoscopic sinus surgery (ESS) is advised. It is still not clear what extension of ESS is required and whether frontal sinusotomy or ethmoidectomy is justified in ODS with frontal sinus involvement. **Methods**: Adult patients presented with uncomplicated recalcitrant bacterial ODS due to endodontic-related dental pathology were evaluated by an otolaryngologist and a dentist and scheduled for ESS. Sinus CT scan demonstrated opacification of maxillary sinus and partial or complete opacification of extramaxillary sinuses ipsilateral to the side of ODS. Patients were undergoing either maxillary antrostomy, antroethmoidectomy, or antroethmofrontostomy. Preoperative and postoperative evaluations were done with nasal endoscopy, dental examination, subjective and radiological symptoms. **Results**: The study group consisted of 30 patients. Statistically significant decreases in values after surgery were found for SNOT-22, OHIP-14, Lund–Mackay, Lund–Kennedy, and Zinreich scale. Tooth pain was present in 40% cases during the first visit and in 10% during the follow-up visit. Foul smell was initially reported by 73.3% and by one patient during follow-up visit (3.3%). Significantly longer total recovery time and more crusting was marked for antroethmofrontostomy when compared to maxillary antrostomy. **Conclusions**: ESS resolved ODS with ethmoid and frontal involvement in almost every case. Minimal surgery led to improved overall clinical success in the same way as antroethmofrontostomy without risking the frontal recess scarring and stenosis.

## 1. Introduction

Odontogenic sinusitis (ODS) is the most common cause of unilateral maxillary sinus opacification, causing 45–75% of these cases [1,2]. The therapeutic approach differs from the one of sinusitis without confirmed associated dental pathology [3,4]. An initial course of intranasal steroids and sometimes various courses of antimicrobial therapy is the first line of therapy [5]. In case of persistence of the disease, endoscopic sinus surgery (ESS) is advised to eliminate the origin of the pathology and enable proper sinus drainage and ventilation [4,6,7].

Endodontic disease with formation of periapical lesions (PAL) is considered one of the most common ODS [8,9]. Endodontic infection may be described as bacterial colonization of the root canals, a consequence of pulp exposure caused primarily by caries or dental trauma [8,10,11]. The root apices of posterior maxillary molars are near the floor of the maxillary sinus (MS). Both are frequently separated by only a thin layer of alveolar bone or sinus mucosa. Bone degradation in response to intracanal bacterial infection may result in apical periodontitis with or without formation of periapical lesions [11,12,13,14].

Subsequent inflammation of sinus mucosa may cause the obstruction of natural ostium with pus formation in the maxillary sinus. Edema and pus may be visible in the middle meatus which confirms the infectious sinusitis of odontogenic source [1,2,3]. If left untreated, infection may affect other sinuses of frontal compartment, usually anterior ethmoids and frontal sinus.

The initial goal for the treatment of chronic rhinosinusitis (CRS) is to diminish mucosal edema and improve sinus drainage, relieving symptoms and improving quality of life [15].

Regarding the treatment of ODS, endoscopic sinus surgery (ESS) is highly indicated for intractable disease that requires surgery [1,2,3,4,5,6,7], but optimal timing of ESS and whether intractable ODS patients undergo primary dental treatment, ESS or both is still controversial [16]. Studies on primary dental treatment for ODS have demonstrated an average 60% success rate, mainly with extractions for apical periodontitis [17]. Extramaxillary spread of the disease was proved to be one of the reasons of unsuccessful resolution of symptoms, and more often requires ESS [9,17,18,19].

Primary ESS is advised in ODS patients with high sinusitis symptom burdens and in those with extramaxillary sinus opacification on CT, as the time to treatment completion and sinusitis resolution was faster than in those patients, who had received only a dental treatment [7,17].

The extension of ESS in surgical treatment for ODS is still poorly described in the literature. This condition requires unique diagnostic criteria and a treatment regimen that differs from nonodontogenic rhinosinusitis [20,21]. At first, some authors suggested that ODS treatment should focus not only on the maxillary sinus but also on all sinonasal cavities involved to minimize the number of recurrences [20]. The majority of published studies up to date do not report the ESS extension for ODS to resolve completely [22,23,24].

Patients undergoing ethmoidectomy, or especially frontal sinusotomy as a part of their surgical treatment, have had a relatively high complication rate. As extensive granulation tissue is frequently encountered in ODS, when combined with bleeding from the highly inflamed mucosa, increases the risk of frontal recess mucosal damage and postoperative crusting, fibrosis, and eventually stenosis [22].

On the other hand, histopathological studies suggested that despite severe inflammation, the ciliated columnar epithelium of intractable ODS is not irreversibly injured and mucocilliary function can recover once the ventilation and drainage of the MS is successfully restored [25].

Thus, it is still not clear what extension of ESS is required to resolve the ODS completely and whether frontal sinusotomy or ethmoidectomy is justified.

### Aim

The aim of the study was to assess if maxillary antrostomy alone, or maxillary antrostomy with anterior ethmoidectomy is sufficient to resolve the extramaxillary sinus disease in ODS with periapical lesions with inflammatory process reaching ethmoid and frontal sinuses.

The study questions to answer were also: does minimal surgery lead to improved overall clinical success and was it faster when compared to antroethmofrontosomy?

Did additional variables such as dental pain, foul smell and endoscopic findings influenced also the final surgical treatment success rate?

## 2. Materials and Methods

The study was conducted in the tertiary referral center during the years 2020–2023. Adult patients who presented with uncomplicated bacterial ODS due to endodontic-related dental pathology (apical periodontitis with or without periapical lesions on imaging, prior failed root canal therapy (RCT)) and had had definitive dental treatment either prior to ESS or after ESS were included to this prospective cohort study.

All patients were consulted by otolaryngologists and dental specialists to confirm ODS.

Sinus CT (or CBCT) scans of included patients demonstrated at least partial or complete opacification of maxillary sinus and either partial or complete opacification of extramaxillary sinuses ipsilateral to the side of ODS (anterior ethmoid cells and frontal sinus). Periapical lucency around maxillary molars and/or premolars greater than 2 mm was considered pathological when combined with clinical symptoms or after prior failed RCT.

**Figure 1:**
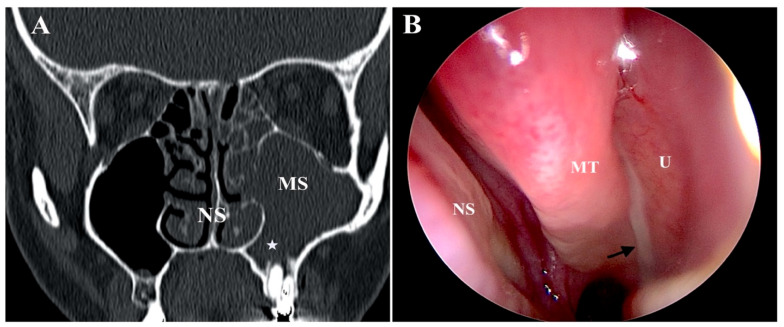
Periapical lesion with alveolar bone loss (marked with an asterisk) and total opacification of maxillary sinus (MS) with an extension to the anterior ethmoids visible on coronal CT scan (**A**) and pus in the middle meatus visible during nasal endoscopy (**B**); pus marked with a black arrow. NS—nasal septum, MT—middle turbinate, U—uncinate process.

The otolaryngologist assessed via nasal endoscopy for mucopurulence or edema in middle meatus or sinuses on endoscopy, which was regarded as confirmation of infectious sinusitis. The dental specialist assessed the pulp vitality with cold pulp testing [8], and checked the tooth with percussion, palpation and mobility test. An example of clinical presentation on radiological and endoscopic images is shown in Figure 1.

The subjective symptoms were also assessed with the use of SNOT-22 and OHIP-14 questionnaires.

SNOT-22 is a validated tool commonly used to assess quality of life. It includes 22 questions, and the score of each question ranges from 0 to 5, with 5 being the worst. Higher scores represent lower health-related quality of life. A score change of at least 8.9 is considered the minimally important difference (the smallest change that can be detected by a patient) in the clinical setting [26,27].

OHIP-14 is an instrument to measure the impact of oral health problems on quality of life. It has a 5-point Likert-type response scale (0: never, 1: hardly ever, 2: occasionally, 3: fairly often, 4: very often) and was validated for the Polish language [28].

The following demographic and clinical variables were recorded: age, gender, ODS laterality, sinusitis symptoms, and nasal endoscopy findings (purulence, edema, polyps) at initial and final visits; sinus opacification extents on computed tomography (CT); additional symptoms such as foul smell and dental pain were also noted.

On the onset of surgery primary ESS extents were noted. Patients were undergoing either maxillary antrostomy only (A), antrostomy and anterior ethmoidectomy (A + E) or maxillary antrostomy, ethmoidectomy (anterior or total), combined with frontal sinusotomy (Draf IIA or IIB procedures), marked as A + E + F. In the latter cases, a 70-degree endoscope was used, and the widest possible opening was created from nasal frontal beak to skull base and from lamina papyracea to middle turbinate [29].

All sinuses with pus (defined as the presence of opaque/colored mucus) got irrigated with at least 100 mL isotonic saline.

Postoperative antibiotics were administered for 7–10 days. Suggested dental treatments performed either before or after ESS and the need for tooth extraction was also noted.

The exclusion criteria were: patients who had undergone prior endoscopic sinus surgery; complicated ODS (with extrasinus spread); maxillary sinus with only mucosal thickening on sinus CT without either clinical or endoscopic symptoms (as it is no longer regarded an infectious sinusitis); and sphenoid sinus opacification on CT or bilateral sinusitis (even with coexistence of bilateral periapical lesions), as it was proven to have reverse correlation with ODS.

Additionally, the patients with sinonasal neoplasm, autoimmune disease, and primary or acquired immunodeficiency and concomitant non-odontogenic rhinosinusitis (fungal, non-odontogenic CRS, maxillary sinus atelectasis, antrochoanal polyp) were excluded from the study.

### 2.1. Data and Outcome Measures

For all patients data on age, gender and medical comorbidities such as allergic rhinitis, asthma, diabetes mellitus and active smoker status were gathered. Overall score in SNOT-22 and OHIP-14 questionnaires preoperatively and postoperatively was noted, and separately the presence or absence of foul smell and dental pain.

Preoperative and postoperative CT grading of each sinus according to the Lund–Mackay scale was performed (maxillary, anterior ethmoids, posterior ethmoids, frontal, and sphenoid sinuses are each graded between 0 and 2, with the meaning of 0 = no abnormality; 1 = partial opacification; 2 = total opacification and ostiomeatal complex is graded either 0 = no abnormality or 2 = total opacification). Maxillary sinus was additionally scored with the use of the Zinreich scale.

The *European Position Paper on Rhinosinusitis and Polyps 2020* considers a total Lund–Mackay score of 2 to be clinically significant when due to complete obstruction of 1 sinus; any total score of 3 or greater is clinically significant, whether from unilateral or bilateral components [15].

Endoscopy findings intraoperative and postoperative were scored with the use of the Lund–Kennedy scale, and the presence of edema, discharge, polyps, crusting, and scarring was noted.

First follow-up appointments were arranged within 7–10 days, and second were arranged for around 4–8 weeks after surgery. Additional follow-up visits were performed as needed around 3–6 months after surgery.

Time to total resolution of symptoms was calculated as days from surgical intervention, with resolution defined as: SNOT-22 decrease by ≥9 [26], decrease in OHIP-14 questionnaire (reports suggested an MID of 5 points using the OHIP-14 for routine dental care in patients undergoing various dental treatments) [30], no pus on nasal endoscopy, and absent foul smell.

The need for dental extraction either prior to or after ESS was also marked.

### 2.2. Statistical Analysis

First, descriptive statistics of the analyzed variables were presented: in the case of quantitative variables, means ± SD, median and range, and—in the case of qualitative variables—frequency and percentage distribution.

The non-parametric Wilcoxon paired test was used to check whether there was a statistically significant change in the Lund–Mackay, Lund–Kennedy, and Zinreich scale scores before and after the procedure, as well as a change in the subjective assessment of symptoms on the SNOT-22 and OHIP-14 scales.

The non-parametric Kruskal–Wallis test was used to compare the recovery time, Lund–Mackay, Lund–Kennedy, and Zinreich scale scores between groups. Moreover, if statistically significant differences were found, it was checked between which pairs of groups they occurred. Due to multiple comparisons, Bonferroni corrections were applied, and the level of statistical significance in this case was set at α = 0.025.

To check whether there is a relationship between the occurrence of scarring and crusting and the method of surgery, the chi-square test and Fisher’s exact test were used, respectively. The chi-square test was also used to check whether there was no statistically significant difference between the groups due to gender, the presence of comorbidities and the side operated on.

The level of statistical significance was set at α = 0.05.

### 2.3. Statement of Ethics

The study was conducted in accordance with the Declaration of Helsinki and approved by the Institutional Ethics Committee of Military Institute of Medicine (protocol No. 43/WIM/2019, permission given in November 2019) for studies involving humans.

## 3. Results

At first, 33 patients were included, but 3 did not show up on the second follow-up visit and were therefore excluded from the analysis. The final study group consisted of 30 patients, mean age 49.2 ± 11.9 years, median 46 years, age range from 30 to 75 years. There were 13 females (43.3% of the group) and 17 males (56.7% of the group). Regarding the surgical treatment, patients were randomly divided into three groups: in Group A, only middle meatal antrostomy (A) was performed (9 people, 3 women, and 6 men): mean age 42.2 ± 9.5 years, median 39 years, age range from 30 to 60 years, in group A + E the antroethmoidectomy was performed (11 people, 5 women, and 6 men) mean age 55.1 ± 9.6 years, median 55 years, age range 41 to 69 years. In the last group (A + E + F) antrofrontoethmoidectomy was performed (10 subjects, 5 females, and 5 males) mean age 49.1 ± 13.4 years, median 46.5 years, age range 31 to 75 years.

For each of the subgroups, pre- and post-treatment assessments were made using the Lund–Mackay, Lund–Kennedy, and Zinreich scales. In addition, the change in subjective symptoms was checked using the SNOT-22 and OHIP-14 scales. Moreover, the number of crusting and scarring was compared in patients from group A + E + F with those in groups A and A + E. It was also checked whether there was a statistically significant difference in recovery time depending on the procedure performed. The descriptive statistics of the abovementioned parameters is given in Table 1.

There was no statistically significant difference between groups A, A + E and A + E + F in the distribution of gender, comorbidities, and the operated side. The exact data are shown in Table 2.

**Figure 2:**
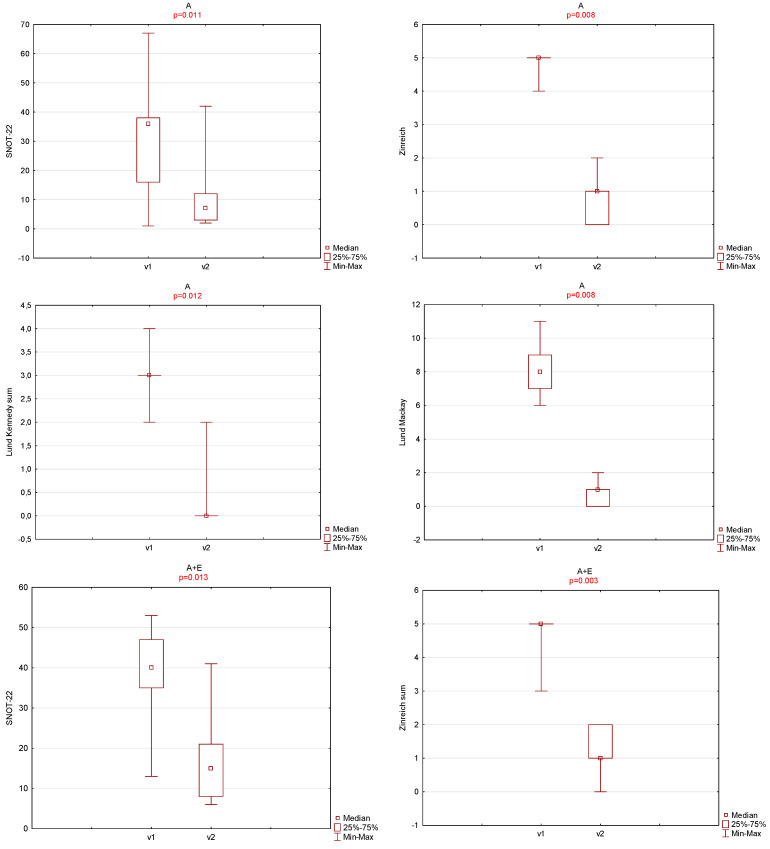

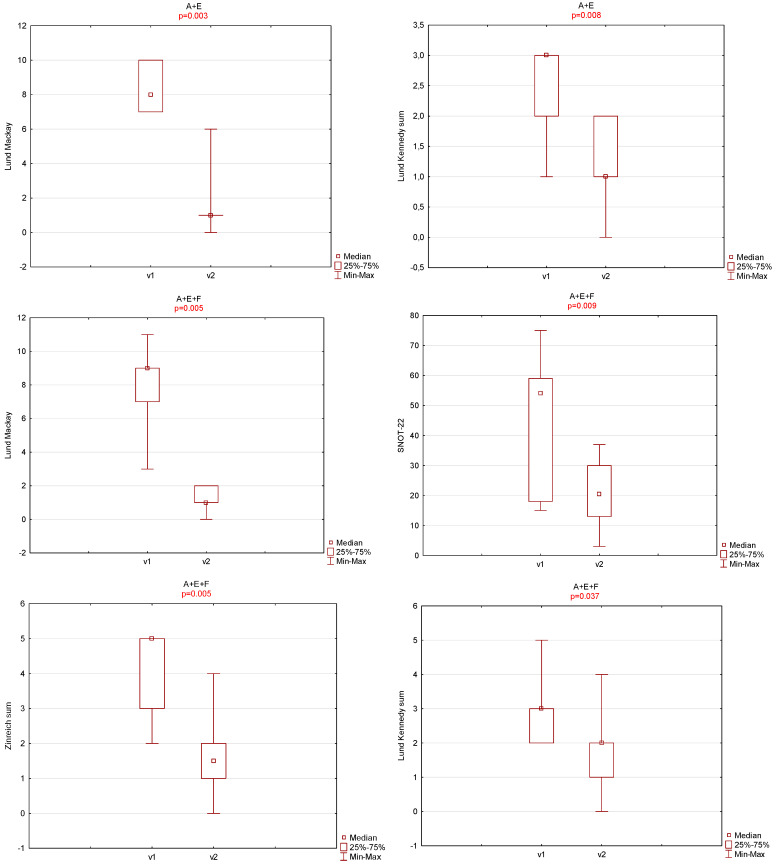
SNOT-22, OHIP-14, Lund–Mackay, Lund–Kennedy scale, and Zinreich scale results for each treatment group during the first (v1) and last (v2) otolaryngological visits.

In case of each group, a statistically significant decrease in values after surgery was found for each of the analyzed parameters. The obtained results were illustrated graphically on Figure 2.

**Figure 3:**
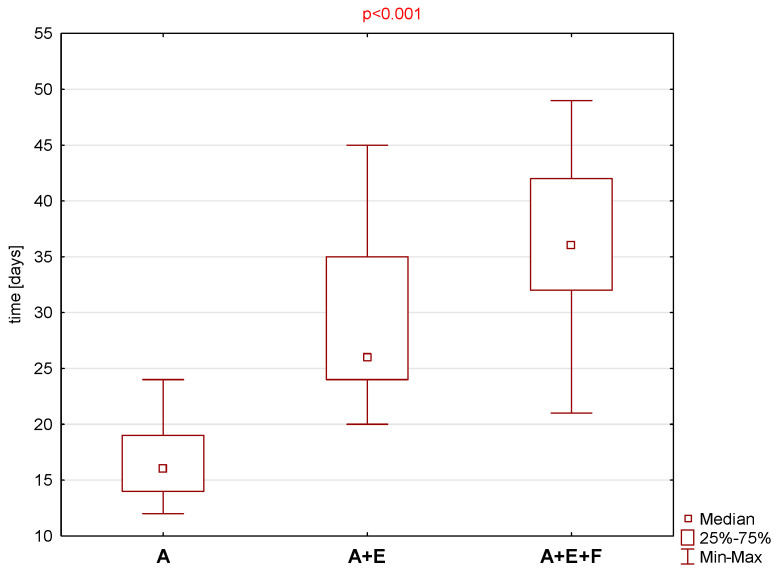
Total time to resolution in group A, A + E and A + E + F with median, range, and the *p* value.

A statistically significantly longer total recovery time was found between groups of patients in which the antroethmofrontostomy (A + E + F) was performed when compared to the group in which the middle meatal antrostomy was performed. When total time to resolution in Group A + E + F was compared to Group A + E, the *p* = value was equal to 0.072. The results are illustrated graphically in Figure 3.

Although there was no statistically significant difference in the occurrence of scarring between the groups, significantly more crusting occurred in the A + E + F group during the control visit when compared to the A and A + E groups. The results are given in Table 3.

**Figure 4:**
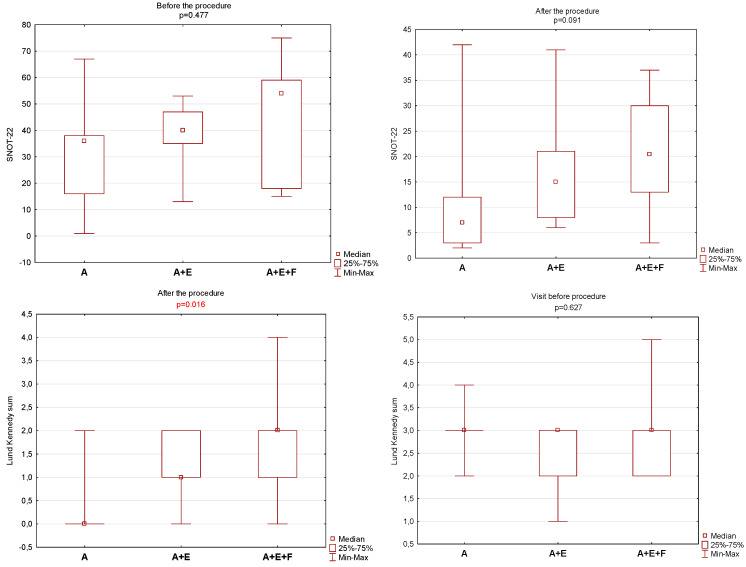
Comparison between pre- and post-treatment visit groups in different extent of sinus surgery (A—antrostomy, A + E—antroethmoidectomy, A + E + F—antroethmofrontostomy) and *p*-value for changes in SNOT-22 overall score and Lund-Kennedy overall score.

In order to check whether there is a statistically significant difference in the results on the Lund–Mackay, Lund–Kennedy, and Zinreich scales between the groups before and after the procedure, the non-parametric Kruskal–Wallis test was used. Before the procedure, there was no statistically significant difference between the groups for any of the scales. After the procedure, statistically significant differences were found only in the Lund–Kennedy scale. The exact data are given in Figure 4.

Tooth pain was reported by 12 out of 30 patients (40%) during the first visit, and in 10% during follow-up visit. Foul smell was initially reported by 22 out of 30 patients (73.3%) and by 1 patient during follow-up visit (3.3%). In the latter case the ODS was considered not resolved completely and based on CT and endoscopy patient was planned for a revision surgery.

The exact values are given in Table 4.

There was no statistically significant difference in the occurrence of dental pain between the groups, but the foul smell was correlated with the higher scores on the radiological Zinreich scale (*p* = 0.031) and the Lund–Kennedy scale (*p* < 0.001).

During the follow-up, in order to resolve ODS completely, 7 out of 9 patients in Group A required tooth extraction after ESS (77.8%)—54.5% for Group A + E and in 60% for Group A + E + F. The fact of postoperative tooth extraction not affected the time to total resolution of symptoms in statistically significant way (*p* = 0.328).

## 4. Discussion

Collaboration between otolaryngologists and dental specialist is essential for optimal treatment of ODS [1,3,4,8]. The therapeutic algorithm usually includes two stages: conservative (antibiotic treatment, endodontic therapy, nasal steroids and decongestants) and surgical. The aim of the treatment is to eliminate the underlying inflammatory cause, relieve symptoms and restore the normal function of the sinus [21].

If the underlying cause is inflammation in the periapical area, RCT, endodontic retreatment or endodontic microsurgery are possible ways of management [10,31,32]. The success rate of initial endodontic therapy varies between 53 and 98%, as reported in studies conducted for the first attempt [33], but there are still very few data on RTC success in clinically confirmed ODS with periapical lesions [32].

If during the follow-up visits there are no signs of healing or if the symptoms persist, surgical therapy may be required [21,33]. Endoscopic sinus surgery has been considered the treatment of choice [3,34]. 

The initial goal for the treatment of CRS without nasal polyps is to reduce mucosal edema and improve sinus drainage, with symptoms reduction, and improvement of quality of life [15]. In non-odontogenic sinusitis, all the sinuses should be addressed during ESS if possible in order to restore to proper ventilation and mucociliary transport in sinonasal mucosa. Taking into consideration that ODS differs in pathophysiology and management from rhinogenic chronic sinusitis, extension of ESS is still unclear [4,35,36].

Early studies that reported the incidence of extramaxillary sinus extension have encouraged surgical treatment of all involved sinuses [20,23,37].

In a study by Crovetto-Martinez et al. [23] half of the patients with an odontogenic maxillary sinusitis presented with an anterior ethmoiditis. The healing of ODS by ESS was achieved in 94.5% after the first intervention and ethmoid involvement does not worsen the overall surgical results although in case of ethmoiditis the affected sinuses were all surgically opened.

There are only two prospective studies up to date that have analyzed the ODS resolution with frontal involvement after middle meatal antrostomy only [22,38]. The maxillary, frontal, and anterior ethmoid sinuses were involved in each case, and patients underwent maxillary middle meatal antrostomy alone. The conclusion of published studies was that frontal sinusitis will resolve once the dental infectious process has been eliminated and the MS drained.

In each group of patients in the presented cohort, a statistically significant decrease in SNOT-22 score, and radiological and endoscopic symptoms after surgery was found despite the varying extension of ESS. In the group with antrostomy, anterior ethmoidectomy, and frontostomy performed, time to total resolution of symptoms was significantly longer, more crusting in nasal cavities was observed, and less radical radiological resolution was marked during the follow-up visits in comparison to patients that had undergone the minimally invasive surgery. This is in accordance with assumptions that pus andgranulation tissue that is frequently encountered in ODS, together with bleeding from inflamed mucosa—increases the risk of frontal recess mucosal damage and postoperative fibrosis and stenosis [22].

The frontal sinusitis reflects a reactive process that regresses spontaneously once the underlying odontogenic condition is addressed and a middle meatal antrostomy had been performed [38], and performing frontal surgery in clinically confirmed ODS is contraindicated.

Foul smell was initially reported by 73.3% of patients and by one patient (3.3%) during the follow-up visit. In the latter case, the ODS was considered not completely resolved, and based on CT scans and nasal endoscopy, the patient was planned for a revision surgery.

In the previously published prospective research [22,38], study groups were relatively small, and the authors did not report the different types of underlying dental disease leading to ODS. 

Although the presented study analyzed the similar number of patients, it focused only on the group that presented for ODS of endodontic origin with periapical lesions that were clinically confirmed by both otolaryngologists and dental specialists.

Endodontic disease and periapical lesions are one of the most common treatable dental pathologies in ODS pathophysiology and proper dental treatment either before or after ESS is strongly encouraged in recalcitrant cases requiring surgical approach [4,7,17,39].

If RCT or retreatment is not possible or the patient did not consent, causative tooth extraction is the final dental treatment of choice [40,41]. During the follow-up in presented cohort, in order to resolve ODS completely 77.8% of patients required tooth extraction after antrostomy, 54.5% after antroethmoidectomy, and 60% after antroethmofrontostomy. The fact of postoperative tooth extraction did not affect the time to total resolution of symptoms, however.

Dental pain in ODS is also not yet thoroughly studied. Tooth pain was reported by 12 out of 30 patients (40%) during the first visit and in 10% during the follow-up visit. It was suggested that when the ventilation and drainage of the MS is successfully restored after ESS, PALs, and odontogenic infection will lead to silent chronic lesions using only antibiotic therapy alone and most causative teeth can be preserved [16,25]. There is a need for further research to confirm that, but ESS preceding dental treatment or extraction of the causative tooth might be indicated for intractable ODS scheduled for surgery, leading to faster symptoms resolution [7,16,17].

## 5. Conclusions

Surgical treatment with endoscopic sinus surgery resolved odontogenic sinusitis of endodontic origin with ethmoid and frontal involvement in almost every case. Nevertheless, significantly longer total recovery time, worse radiological resolution of symptoms, and more crusting in postoperative nasal endoscopy were found in group of patients undergoing antroethmofrontostomy when compared to the group in which the middle meatal antrostomy was performed.

Minimal surgery led to improved overall clinical success in the same way as antroethmofrontostomy without risking the frontal recess scarring and stenosis.

Foul smell was correlated with higher scores in radiological scales initially, but except one case, was resolved after surgical treatment.

## 6. Limitations

Limitations of the study need to be discussed. First, periapical lesions might respond to root canal treatment, and milder sinusitis can resolve after the tooth is properly treated. Although patients included in the study were all evaluated by dental specialists and the RCT was advised, some of them opted for the dental treatment after the ESS procedure. In that case, the data on exact material or technics of endodontic treatment are missing.

Our present study population was relatively small but focused on one specific subgroup of ODS patients. Further prospective studies on larger population will be beneficial regarding different clinical scenarios of treatable and non-treatable dental pathologies of causative teeth.

Additionally, excluding posterior ethmoid and sphenoid involvement on ODS disease might create bias and underestimate the total prevalence of the condition, but multiple studies already have shown that these sinuses are rarely involved or even reversely correlated with ODS prediction [2,3,8,28,42].

## Figures and Tables

**Table 1:** Descriptive statistics of total cohort and each treatment group separatively (A—antrostomy, A + E—antroethmoidectomy, A + E + F—antroethmofrontostomy).

	OHIP-14	SNOT-22	Zinreich Scale Overall	Lund Kennedy Overall	BMI	Time to Total Recovery (Days)
**First visit**	***Groups A, A + E, A + E + F n = 30***					
**Mean ± SD**	11.1 ± 11.0	38.8 ± 19.1	4.5 ± 0.9	2.8 ± 0.8	26.6 ± 5.0	28.0 ± 10.4
**Median** **(Min–Max)**	7 (0–46)	37.5 (1–75)	5 (2–5)	3 (1–5)	25.0 (18.4–40.5)	26.0 (12.0–49.0)
**Second visit**						
**Mean ± SD**	5.0 ± 5.4	16.9 ± 11.8	1.3 ± 0.9	1.1 ± 1.1	n/a	n/a
**Median** **(Min–Max)**	3.5 (0–18)	13.5 (2–42)	1 (0–4)	1 (0–4)	n/a	n/a
**First visit**	***Group A n = 9***					
**Mean ± SD**	6.2 ± 3.7	31.7 ± 22.6	4.8 ± 0.4	2.9 ± 0.6	26.3 ± 6.5	16.9 ± 3.9
**Median** **(Min–Max)**	7 (0–12)	36 (1–67)	5 (4–5)	3 (2–4)	24.5 (18.4–40.5)	16 (12–24)
**Second visit**						
**Mean ± SD**	3.4 ± 5.8	11.8 ± 13.0	0.9 ± 0.8	0.3 ± 0.7	n/a	n/a
**Mediana** **(Min–Max)**	1 (0–18)	7 (2–42)	1 (0–2)	0 (0–12)	n/a	n/a
**First visit**	***Group A + E n = 11***					
**Mean ± SD**	10.0 ± 9.4	39.0 ± 10.9	4.7 ± 0.6	2.5 ± 0.7	27.8 ± 4.8	29.9 ± 8.2
**Median** **(Min–Max)**	7 (0–29)	40 (13–53)	5 (3–5)	3 (1–3)	26.7 (22.1–37.2)	26 (20–45)
**Second visit**						
**Mean ± SD**	6.4 ± 6.6	17.6 ± 10.9	1.3 ± 0.6	1.2 ± 0.8	n/a	n/a
**Median** **(Min–Max)**	5 (0–18)	15 (6–41)	1 (0–2)	1 (0–2)	n/a	n/a
**First visit**	***Group A + E + F n = 10***					
**Mean ± SD**		45 ± 22.2	4.1 ± 1.3	2.9 ± 1.0	25.6 ± 3.9	35.8 ± 7.9
**Median** **(Min–Max)**		54 (15–75)	5 (2–5)	3 (2–5)	25.3 (18.7–31.0)	36 (21–49)
**Second visit**						
**Mean ± SD**		20.6 ± 11.0	1.6 ± 1.1	1.7 ± 1.3	n/a	n/a
**Median** **(Min–Max)**		20.5 (3–37)	1.5 (0–4)	2 (0–4)	n/a	n/a

Mean and standard deviation (±SD), median and range data for Oral Health Impact Profile-14 (OHIP-14), Sino-Nasal Outcome Test-22 (SNOT-22), Zinreich and Lund–Kennedy scale as well as body mass index (BMI) and time to total recovery are given for the first and last otolaryngological visit. n/a- not applicable.

**Table 2:** Frequency and percentage distribution of gender; affected side of sinuses and comorbidities in each treatment group (A—antrostomy, A + E—antroethmoidectomy, A + E + F—antroethmofrontostomy).

Groups	Gender *p* = 0.753		Affected Side *p* = 0.679		
	Female	Male	Right		Left
**A**	3 (33.3%)	6 (66.7%)	7 (77.8%)		2 (22.2%)
**A + E**	5 (45.5%)	6 (54.5%)	8 (72.7%)		3 (27.3%)
**A + E + F**	5 (50%)	5 (50%)	6 (60%)		4 (40%)
	**Comorbidities *p* = 0.396**				
	None reported	Asthma	Allergy	Diabetes	Active Smoker
**A**	6 (66.7%)	0 (0%)	0 (0%)	0 (0%)	3 (33.3%)
**A + E**	5 (45.4%)	1 (9.1%)	0 (0%)	1 (9.1%)	4 (36.4%)
**A + E + F**	6 (60%)	0 (0%)	2 (20%)	0 (0%)	2 (20%)

**Table 3:** Extent of sinus surgery in each treatment group (A—antrostomy, A + E—antroethmoidectomy, A + E + F—antrofrontoethmoidectomy) with percentage distribution of crusting and scarring and respective *p*-value.

Extent of Sinus Surgery	Crusting—0	Crusting—1	*p*-Value	Scarring—0	Scarring—1	*p*-Value
**A**	9 (100%)	0	<0.001	8 (88.9%)	1 (11.1%)	0.363
**A + E**	11 (100%)	0		8 (72.7%)	3 (27.3%)	
**A + E + F**	4 (40%)	6 (60%)		6 (60%)	4 (40%)	

**Table 4:** Presence or absence of dental pain and foul smell with corresponding level of statistical significance (*p* value) in each treatment group and overall. A—antrostomy, A + E—antroethmoidectomy, A + E + F—antrofrontoethmoidectomia, V1—before surgical treatment, V2—after treatment.

*Extent of Surgical Treatment*	Dental Pain (0—Absent/1—Present)				*p*-Value
	V1		V2		
	0	1	0	1	
***A***	7 (77.8%)	2 (22.2%)	9 (100%)	0 (0%)	*p* = 0.471
*A + E*	7 (63.6%)	4 (36.4%)	8 (72.7%)	3 (27.3%)	*p* = 0.987
*A + E + F*	4 (40%)	6 (60%)	10 (100%)	0 (0%)	*p* = 0.011
*Overall*	18 (60%)	12 (40%)	27 (90%)	3 (10%)	*p* = 0.0153
***Extent of Surgical Treatment***	**Foul Smell (0—Absent/1—Present)**				***p*-Value**
	**V1**		**V2**		
	**0**	**1**	**0**	**1**	
*A*	1 (11.1%)	8 (88.9%)	9 (100%)	0 (0%)	*p* < 0.001
*A + E*	3 (27.3%)	8 (72.7%)	10 (90.9%)	1 (9.1%)	*p* = 0.001
*A + E + F*	4 (40%)	6 (60%)	10 (100%)	0 (0%)	*p* = 0.011
*Overall*	8 (26.7%)	22 (73.3%)	29 (96.7%)	1 (3.3%)	*p* < 0.001

## Data Availability

The raw data supporting the conclusions of this article will be made available by the corresponding author on request.
